# Authors’ Response to Peer Reviews of “Cardiotoxicity in Pediatric Cancer Survivorship: Retrospective Cohort Study”

**DOI:** 10.2196/79672

**Published:** 2025-07-31

**Authors:** Masab Mansoor, Andrew Ibrahim

**Affiliations:** 1 Edward Via College of Osteopathic Medicine, 4408 Bon Aire Drive, Monroe, LA, 71203, United States, 1 504-521-3500; 2 Texas Tech University Health Sciences Center School of Medicine, Lubbock, TX, United States

**Keywords:** cardiotoxicity, cardiology, cardiovascular, heart, arrhythmias, self-reported questionnaires, oncology, survivors, pediatrics, prevalence, incidence, risk, epidemiology, anthracycline exposure, childhood cancer survivors

*This is the authors’ response to peer-review reports for “Cardiotoxicity in Pediatric Cancer Survivorship: Retrospective Cohort Study.”*

## Round 1 Review

### Reviewer ET [1]

#### General Comments

*This paper [* *2* *] gives valuable insights into cardiotoxicity in pediatric cancer survivorship: patterns, predictors, and implications for long-term care. The results and methodology are sound. However, some minor revisions would improve clarity and strengthen the overall impact of this paper. Below are my suggestions.*

#### Major Comments

*1. Method section (study population and data source): In the Method section, specifically the fourth line, the description “of 21 at one of 31 participating institutes” is unclear. The sentence should be revised for better clarity.*

**Response:** We thank the reviewer for highlighting this lack of clarity. We have revised this sentence for better clarity as follows: “Eligible participants were those diagnosed with cancer before the age of 21 years who were treated at one of the 31 participating institutions across the United States and Canada. These institutions collectively represent major pediatric oncology centers providing comprehensive coverage across North America” (page 6, Methods section).

*2. Missing answer for seventh objective: The answer to the seventh objective is unclear.*

**Response:** We appreciate this important observation. We have significantly expanded the section on cardioprotective factors (objective 7) in the Results section to provide a more comprehensive and clear answer. We have included detailed information about protective associations identified in our analysis, including physical activity, cardioprotective medications, dexrazoxane administration, and nutritional factors, along with specific hazard ratios and CIs for each (page 14, Results section).

#### Minor Comments

*1. Result presentation: It would be better if the results were presented in tabular format for easier comprehension. A table would help summarize the key findings and increase readability.*

**Response:** We agree with this suggestion and have added two new tables to present our results more clearly:

- Table 1: Demographic and clinical characteristics of childhood cancer survivors (page 9).
- Table 3: Risk factors for cardiovascular complications in childhood cancer survivors (page 12).

These tables complement the existing Table 2 (summary of key findings) and provide a more comprehensive visualization of our results.

*2. Clarity in results numbering: To improve clarity, it would be beneficial to present all the results with corresponding numbers, matching each result with the respective objective number for easier reference and alignment.*

**Response:** Following this helpful suggestion, we have reorganized our Results section to clearly number each subsection according to its corresponding objective. The Results section now features the following structure:

- Study Population Characteristics (Background to All Objectives)
- Incidence of Cardiovascular Complications (Objective 1)
- Temporal Patterns and Treatment Era Effects (Objectives 2 and 4)
- Risk Factors for Cardiovascular Complications (Objective 3)
- Risk Prediction Model (Objective 5)
- Impact on Survival and Quality of Life (Objective 6)
- Exploration of Cardioprotective Factors (Objective 7)
- Comparison with Sibling Controls (Objective 8)

This organization ensures a direct alignment between our stated objectives and the presentation of our results.

### Reviewer FS [3]

*The study relies heavily on self-reported cardiovascular complications, which may introduce reporting bias. While a subset of cases was validated via medical records, the proportion of validated cases is not explicitly stated, and the possibility of underreporting or overreporting remains. The reliance on self-reported cardiovascular complications may have introduced reporting bias into the study. Although some cases were validated through medical records, the proportion of validated cases remains unclear, leaving the potential for underreporting or overreporting. The authors could also consider exploring linkage with external databases (eg, insurance claims, hospital records) for additional validation.*

**Response:** We acknowledge this important limitation. In our revised manuscript, we have explicitly stated that 27% of all self-reported cardiovascular events were confirmed through medical record review, with a confirmation rate of 93% for self-reported cardiovascular conditions (page 7, Methods section). Additionally, we have expanded our discussion of this limitation in the “Strengths and Limitations” section, noting that we conducted sensitivity analyses restricted to medically confirmed cases, which yielded similar results (page 16, Discussion section).

*The manuscript presents a risk prediction model (C statistic 0.78), but there is no external validation or discussion of its clinical applicability. Validate the model using an independent dataset (eg, a subset of Childhood Cancer Survivor Study data withheld from model training or another survivor cohort). Report calibration metrics (eg, Hosmer-Lemeshow test, calibration plots) to assess model accuracy. Provide a clinical risk score or decision framework for practical implementation.*

**Response:** We appreciate this insightful comment. We have expanded our discussion of the risk prediction model to address the lack of external validation, noting that this was not feasible due to the lack of comparable cohorts with similar long-term follow-up. However, we have provided additional details on internal validation using bootstrapping techniques and have added information about a simplified risk score system we developed to facilitate clinical application. This scoring system assigns points to key risk factors and identifies survivors at high risk who may benefit from enhanced cardiovascular surveillance (page 13, Results section).

*The study reports a decreasing risk of cardiotoxicity over time, suggesting improvements in treatment protocols. However, this could be confounded by survivor selection bias (eg, patients with higher early mortality due to severe toxicity were less likely to be included in later eras).*

*Adjust for potential survivor bias using inverse probability weighting or sensitivity analyses. Consider comparing treatment regimens (eg, changes in anthracycline dosages, cardioprotective measures) across eras to explicitly determine which interventions contributed to reduced risk. The research indicates that the risk of cardiotoxicity diminishes over time, suggesting that treatment protocols have become more effective. However, it is possible that this observation is attributable to survivor selection bias, wherein patients who succumbed to severe toxicity early in the study were not included in subsequent phases. To address potential survivor bias, researchers should employ methodologies such as inverse probability weighting or sensitivity analyses. Additionally, treatment regimens (eg, modifications in anthracycline dosages and cardioprotective measures) should be compared across different time periods to ascertain which interventions are responsible for the diminished risk.*

**Response:** We thank the reviewer for this astute observation. We have addressed this concern in the Discussion section by acknowledging that the observed trend of decreasing cardiovascular risk across treatment eras might be partially influenced by survivor selection bias. We have described sensitivity analyses using inverse probability weighting to account for potentially informative censoring, which yielded similar, albeit slightly higher, risk estimates. Additionally, we have noted our comparison of treatment protocols across eras, which found that reductions in anthracycline doses and implementation of cardiac-sparing radiation techniques likely contributed to the genuine reduction in cardiovascular risk in more recent cohorts (page 15, Discussion section).

*The study focuses on clinically evident cardiovascular complications but does not assess subclinical cardiotoxicity, which could be detected via biomarkers or imaging.*

*Incorporate cardiac biomarkers (eg, troponins, N-terminal pro–brain natriuretic peptide) in a subset of survivors to identify early signs of myocardial damage. Perform echocardiographic or cardiac magnetic resonance imaging evaluations in a subgroup to detect preclinical cardiac dysfunction. This could strengthen the study’s ability to recommend early intervention strategies.*

*The authors appropriately point out the opportunity to improve early intervention by identifying a subset of survivors for early myocardial damage using cardiac biomarkers and imaging. While this is not possible in the present study, future studies incorporating this approach would allow for detection of subclinical cardiotoxicity.*

**Response:** We agree with this limitation and have expanded our discussion to acknowledge that our study focused on clinically evident cardiovascular complications and did not assess subclinical cardiotoxicity. We have noted that the prevalence of subclinical cardiac dysfunction is likely higher than the reported clinically apparent complications and have stated that future studies incorporating cardiac biomarkers and advanced imaging techniques would enable earlier detection of cardiac damage and potentially identify opportunities for preventive interventions before clinical manifestation (page 16, Discussion section).

*The manuscript discusses risk factors but does not evaluate protective factors (eg, exercise, angiotensin-converting enzyme inhibitors, β-blockers). Analyze whether lifestyle modifications (eg, regular exercise) or cardioprotective medications influence the incidence of cardiotoxicity. Conduct a subgroup analysis on survivors who received cardioprotective interventions versus those who did not.*

**Response:** We thank the reviewer for highlighting this gap. We have substantially expanded our Results section to include a comprehensive analysis of cardioprotective factors (objective 7), including physical activity, cardioprotective medications (angiotensin-converting inhibitors, β-blockers, statins), dexrazoxane administration, and nutritional factors. For each of these, we have provided specific hazard ratios and CIs to quantify their protective effects (page 14, Results section).

*Please indicate whether the proportional hazards assumptions were tested and consider reporting Schoenfeld residuals or time-dependent covariate analyses.*

*Please include more details on how missing data were handled.*

*Were there particular domains of quality of life that were lower among those with cardiovascular complications?*

*Consider adding detailed figure legends to improve readability and refining axis labels in existing figures.*

*A table summarizing key risk factors with adjusted hazard ratios and P values would be beneficial.***Response:** We have addressed the technical concerns raised by adding the following information to our manuscript:

- Clarified that we tested proportional hazards assumptions using Schoenfeld residuals and time-dependent covariate analyses (page 8, Methods section)
- Provided more details on how missing data were handled, noting that we used multiple imputation with chained equations for covariates with missing data (page 8, Methods section)
- Added information about quality of life assessments, specifying that we used the 36-item Short Form Health Survey instrument and noting which domains showed the largest decrements among survivors with cardiovascular complications (page 8, Methods section)
- Enhanced figure legends and axis labels for better readability

We are grateful to both reviewers for their thoughtful and constructive feedback, which has significantly improved the quality and clarity of our manuscript.

## Round 2 Review

### Reviewer FS

*Please state the proportion of cases with cardiovascular events confirmed by medical record review.*

**Response:** We have added the specific number of confirmed cases in the Methods section under “Outcome Measures”: “To enhance validity, 27% of all self-reported cardiovascular events (739 of 2743 cases) were confirmed through medical record review by trained abstractors using standardized protocols.”

*Please discuss the increased cardiotoxicity observed in male survivors. Was this due to treatment or other comorbidities that exacerbated previously subclinical cardiac exposures?*

**Response:** We have added a detailed discussion of this gender disparity in the Discussion section, addressing both treatment-related factors and comorbidities. We note that male survivors received higher cumulative anthracycline doses and chest radiation, but also had higher rates of cardiovascular comorbidities that may have exacerbated subclinical cardiac damage. We also briefly discuss potential biological differences, including the cardioprotective role of estrogen in females.

*Please provide a thoughtful description of how the risk model could be integrated into previously described models and recommendations for cardiac risk groups like the International Late Effects of Childhood Cancer Guideline Harmonization Group.*

**Response:** We have added a paragraph in the “Clinical Implications” section discussing how our risk prediction model could be integrated with the International Late Effects of Childhood Cancer Guideline Harmonization Group framework. We propose a two-step approach that maintains consistency with established guidelines while providing more personalized risk estimates.

*Please standardize the reporting/formatting for data into a table format more typical for manuscript reporting for complication rates, multivariate cox regression, and temporal trends.*

**Response:** We have revised Table 2 to show the number of cases and cumulative incidence for each cardiovascular outcome in a standardized format. We have also created two new tables: Table 4 showing the treatment era analysis and Table 5 comparing outcomes with sibling controls, both with appropriate statistical adjustments.

*Please provide a table or figure for the treatment era analysis.*

**Response:** We have created Table 4 displaying the number of patients, events, cumulative incidence, and adjusted hazard ratios across the three treatment eras (1970s, 1980s, 1990s), with *P* values and trend analysis.

*Please provide a table or figure for the sibling controls comparison. Is this after adjustment for age, gender, etc?*

**Response:** We have created Table 5 showing the comparison between survivors and sibling controls for each cardiovascular outcome, with both age- and sex-adjusted odds ratios and fully adjusted odds ratios.

*The CI of cardiovascular complications in childhood cancer survivors data is shown in a nonstandard stacked bar plot format. Please show as CI curves.*

**Response:** We have completely redesigned Figure 1 to display cumulative incidence curves with 95% CIs (shown as shaded areas) for each treatment era and for all survivors combined, replacing the previous stacked bar plot format.

### Additional Revisions Made in Response to Reviewer Comments From Rounds 1 and 2

#### Selection Bias Discussion

We have added a paragraph addressing potential selection bias in the observed trend of decreasing cardiovascular risk across treatment eras. We describe our sensitivity analyses using inverse probability weighting to account for potentially informative censoring and discuss how changes in treatment protocols likely contributed to genuine risk reduction.

#### Limitations Regarding Outcome Ascertainment

We have expanded the Limitations section to explicitly state that 73% of cardiovascular events relied on self-reported outcomes, and described the sensitivity analyses restricted to medically confirmed cases.

#### Discussion of Subclinical Cardiotoxicity

We have added a paragraph at the end of the “Strengths and Limitations” section acknowledging that our study focused on clinically evident cardiovascular complications and did not assess subclinical cardiotoxicity, which might be detected through biomarkers or advanced imaging techniques.
